# Shikimic acid, a mannose bioisostere, promotes hair growth with the induction of anagen hair cycle

**DOI:** 10.1038/s41598-019-53612-5

**Published:** 2019-11-18

**Authors:** Mira Choi, Soon-Jin Choi, Sunhyae Jang, Hye-In Choi, Bo-Mi Kang, Sungjoo Tommy Hwang, Ohsang Kwon

**Affiliations:** 1 Department of Dermatology, College of Medicine, Ilsan Paik Hospital, Inje University, Gyeong-gi, Republic of Korea; 2 Department of Dermatology, College of Medicine, Seoul National University, Seoul, Republic of Korea; 3 Institute of Human-Environment Interface Biology, Medical Research Center, Seoul National University, Seoul, Republic of Korea; 4 Laboratory of Cutaneous Aging and Hair Research, Biomedical Research Institute, Seoul National University Hospital, Seoul, Republic of Korea; 5 Dr. Hwang’s Hair-Hair Clinic, Seoul, Korea

**Keywords:** Molecular medicine, Preclinical research

## Abstract

Shikimic acid (SA) has recently been found to be a major component of plant stem cells. The exact effects of SA on human hair follicles (HFs) is unknown. The purpose of this study was to examine the effects of SA on hair growth. We investigated the effect of SA on an *in vivo* C57BL/6 mouse model. We examined the expression of mannose receptor (MR), which is a known receptor of SA, in human HFs and the effect of SA on human dermal papilla cells (hDPCs), outer root sheath cells (hORSCs), and on *ex vivo* human hair organ culture. SA significantly prolonged anagen hair growth in the *in vivo* mouse model. We confirmed expression of the MR in human HFs, and that SA increased the proliferation of hDPCs and hORSCs. It was found that SA enhanced hair shaft elongation in an *ex vivo* human hair organ culture. SA treatment of hDPCs led to increased c-myc, hepatocyte growth factor, keratinocyte growth factor and vascular endothelial growth factor levels and upregulation of p38 MAPK and cAMP response element-binding protein levels. Our results show that SA promotes hair growth and may serve as a new therapeutic agent in the treatment of alopecia.

## Introduction

Stem cells have the ability to differentiate into various cells with specific functions depending on the environment; they also have the ability to repeatedly divide and renew^1^. Because of these characteristics, active research is underway on the use of stem cells to restore function to organs and tissues in various diseases; some clinical application has been achieved^2^. In particular, stem cell treatment has been extensively studied and proved effective in various hair loss diseases^3,4^. Till date, stem cells from animal origin have primarily been studied; however, stem cells have also been found to exist in plants^5^. In animals, only tissue regeneration occurs after tissue damage, whereas plants are characterized by their ability to induce the development of new individuals beyond restoration when tissue damage occurs; therefore, plant stem cells play an important role in this regeneration mechanism^5^. Many attempts have been made to apply the regenerative ability of plant stem cells to hair loss treatment, but the precise mechanism has not yet been elucidated^6^.

Shikimic acid (SA, 3,4,5-trihydroxy-1-cyclohexene-1-carboxylic acid, C_7_H_10_O_5_) was originally known as an extract obtained from *Illicium anisatum* (Japanese star anise) and *Illicium verum* (Chinese star anise), but can be obtained from dozens of plants^7,8^. SA is known to have antimicrobial, antioxidant, anti-inflammatory, and analgesic effects, and was used as an essential starting material for industrial synthesis of the antiviral oseltamivir (Tamiflu^®^)^7,8^. Recently, it has been reported that SA is a major component of plant stem cells or callus that induces tissue regeneration when a plant is injured^9^. Research has also been reported on the possibility of SA being applied to the treatment of demyelinating disease by promoting the differentiation of oligodendrocyte precursor cells^10^. SA has shown reprogramming activities in human dermal fibroblast and was found to be effective for tissue regeneration^9^. Based on several reports that tissue regeneration and hair growth are closely related, it can be inferred that SA also has a positive effect on hair growth^11^.

A previous study reported that water-soluble extract of *Illicium anisatum* stimulates mouse vibrissae follicles in organ culture, and GC/MS analysis revealed that the said extract contained SA^12^. It was seen that SA induced mRNA expression of insulin-like growth factor (IGF)-1, keratinocyte growth factor (KGF), and vascular endothelial growth factor (VEGF) in the mouse hair follicle^12^.

It is known that SA acts as a mannose bioisostere of the mannose receptor (MR, CD206), which is found in fibroblasts and keratinocytes^13–15^. MR is a carbohydrate-binding receptor, which is known to have an antigen-presenting function; however, recently, it has been reported that MR may affect cell signaling by participating in the p38 mitogen-activated protein kinase (MAPK)-cAMP response element-binding protein (CREB) pathway^14,16^. Based on previous studies, it is suggested that SA may promote hair growth through the MAPK pathway. However, there is no report on the potential effects of SA on human hair follicles (HFs).

Therefore, in this study, we investigated the effect of SA on hair growth *in vivo*, confirmed using the anagen induction assay in C57BL/6 mice as well as in cultured human dermal papilla cells (hDPCs), outer root sheath cells (hORSCs), and *ex vivo* human HF organ culture.

## Materials and Methods

### Ethics statement

The study protocols were approved by the institutional research board of Seoul National University Hospital (IRB No. H-1806-100-952), and written informed consent was obtained from all subjects. All experimental procedures using human tissues were conducted according to the principles described in the Declaration of Helsinki. The animal study was approved by the Institutional Animal Care and Use Committee (IACUC) at Seoul National University Hospital (IACUC No. 18-0071-S1A0) and all methods were performed in accordance with the relevant guidelines and regulations

### Anagen induction assay in C57BL/6 mice

We performed an anagen induction assay as previously described^17^. The back skin of 7-week-old C57BL/6 female mice in the telogen phase was shaved with a clipper. Vehicle (70% polyethylene glycol + 30% ethanol), SA (Sigma-Aldrich, St. Louis, MO, USA) (10 and 100 mM), and minoxidil (MNX, 2%) were topically applied every weekday for 3 weeks. Skin thickness and anagen induction score were assessed using a modified version of previously described methods^17–19^. On performing histological analysis via H&E staining, skin thickness was measured as the distance from the top of the epidermis to the bottom of the subcutaneous fat using the Image J software. Anagen induction scores were calculated using an assigned arbitrary score (telogen = 1, anagen I-VI = 2–7) and the mean score was compared between mice groups^18^.

### Isolation and culture of HFs

Scalp tissue samples (1.5 × 1.0 cm) from the occipital region were taken from healthy male volunteers without current or prior scalp diseases. The HFs were isolated under a microscope (Olympus, Tokyo, Japan) with forceps, and maintained in William’s E medium (Sigma-Aldrich, St. Louis, MO, USA) at 37 °C in a humidified atmosphere of 95% O_2_ and 5% CO_2_.

### Culture of hDPCs and hORSCs

hDPCs were isolated from dissected HFs and cultured as described previously^19,20^. Simply, candlelight-shaped dermal papilla(DP)s were dissociated and incubated in Dulbecco’s modified Eagle medium (DMEM: Welgene, Daegu, Republic of Korea) supplemented with 10% fetal bovine serum (FBS, Welgene) and 1 × antibiotic/antimycotic solution (Gibco BRL, Gaithersburg, MD, USA) containing amphotericin B, penicillin, and streptomycin at 37 °C in a 5% CO_2_ atmosphere.

For the isolation of hORSCs, the hair shaft and hair bulb region of the HF were cut off to prevent contamination with other cells. After cutting off the hair shaft and hair bulb regions, the trimmed HFs were immersed in DMEM supplemented with 20% FBS. On the 3^rd^ day of culture, the medium was changed to KGM-Gold™ Keratinocyte Growth Medium (Lonza, Walkersville, MD, USA) supplemented with basal medium (bovine pituitary extract, human epidermal growth factor, bovine insulin, hydrocortisone, gentamycin, amphotericin B, epinephrine, and transferrin) at 37 °C in a 5% CO_2_ atmosphere.

### Thiazolyl blue [3-(4,5-dimethylthiazol-2-yl)-2,5-diphenyl tetrazolium bromide] (MTT) assay

To determine the effect of SA on the survival and proliferation of hDPCs and hORSCs, viability was measured using an MTT assay^21^. Cells at 1.0 × 10^4^ cells per well were seeded into a 96-well plate, serum-starved for 24 hours, and then treated with various concentrations of SA for 24, 48 and 72 hours. After adding 20 μL of MTT solution (5 mg/mL) to each well, cells were incubated for 4 hours at 37 °C in the dark and then incubated with 200 μL of dimethyl sulfoxide for 20 minutes at room temperature. The samples were assessed by measuring the absorbance at 570 nm with an enzyme-linked immunosorbent assay reader.

### Human HF organ culture

Human HFs were isolated and cultured as described previously^19,22,23^. Isolated HFs were cut from the bottom of the DP (approximately 3.5 mm in length) and cultured in William’s E medium (Gibco BRL) supplemented with 10 ng/mL hydrocortisone, 10 μg/mL insulin, 2 mM L-glutamine, and 1 × antibiotic/antimycotic solution at 37 °C in a 5% CO_2_ atmosphere. SA was added at final concentrations of 1 and 10 μM. On every third day, elongation of the hair shaft was measured directly using a stereomicroscope (Olympus).

### Immunofluorescence staining

Immunofluorescence staining for CD206 and Ki67 was performed on 5-μm frozen sections of human HFs as previously described^23^. TUNEL labeling (Millipore, Carpinteria, CA, USA) was used as an indicator of cell apoptosis. The antibodies used were anti-Ki67 (DAKO, Carpinteria, CA, USA) and anti-CD206 (Novus Biologicals). A DAPI mounting media kit (Vector Laboratories, Burlingame, CA, USA) was used to counterstain the nuclei.

### Quantitative real-time polymerase chain reaction

Gene expressions were measured as described previously. Briefly, total RNA was isolated from hDPCs using RNA iso Plus (Takara Bio Inc., Otsu, Shiga, Japan) and treated with DNase I (Roche Pharmaceuticals, Welwyn Garden City, UK) to remove genomic DNA. We used 1 μg of total RNA for the cDNA synthesis reaction, which was performed using a First Strand cDNA Synthesis Kit (Fermentas, St. Leon-Rot, Germany) according to the manufacturer’s instructions. To quantitatively estimate mRNA expression, polymerase chain reaction (PCR) was performed on a 7500 Real-Time PCR System (Applied Biosystems, Foster City, CA, USA) using SYBR Premix Ex Taq (Takara Bio Inc.) according to the manufacturer’s instructions. The primer sequences are summarized in Supplementary Table 1. All experiments with SYBR Green were performed in triplicate and were independently repeated more than 3 times. Data are presented as fold change relative to the control normalized to 36B4 expression.

### Western blot analysis

Total protein from hDPCs was extracted using a RIPA lysis buffer (Millipore, Carpinteria, CA, USA) according to the manufacturer’s instructions. Proteins were separated using 10% sodium dodecyl sulfate-polyacrylamide gel electrophoresis and transferred to a polyvinylidene fluoride membrane (Amersham, Buckinghamshire, UK) using a wet transfer system. The blotted membranes were incubated with primary antibodies at 4 °C. The following antibodies were used: anti-total P38 (#9212), anti-phospho P38 (#9211) (Cell signaling Technology, Beverly, MA, USA), anti-phospho CREB (#9198), anti-total CREB (#9197), and anti-α-tubulin (SC-8035) (Santa Cruz Biotechnology, Santa Cruz, CA, USA) antibodies. Membranes were probed with an anti-mouse, anti-rabbit-, or anti-goat-IgG-horseradish peroxidase conjugates (Santa Cruz Biotechnology) for 1 hour at room temperature. Antibody–antigen complexes were detected using the ECL system (Amersham Pharmacia Biotech, Little Chalfont, UK).

### Statistical analysis

All experiments were performed in at least three independent experiments. The paired t-test and Wilcoxon signed rank test were used for statistical analysis. Statistical significance was considered at *P* < 0.05. Repeated-measures ANOVA analysis of variance was used for statistical analysis of data from the organ culture studies. The statistical analyses were done by using the IBM SPSS Statistics 21.0 software package (IBM Co., Armonk, NY, USA).

## Results

### SA stimulates hair growth in C57BL/6 mice

**Figure 1:**
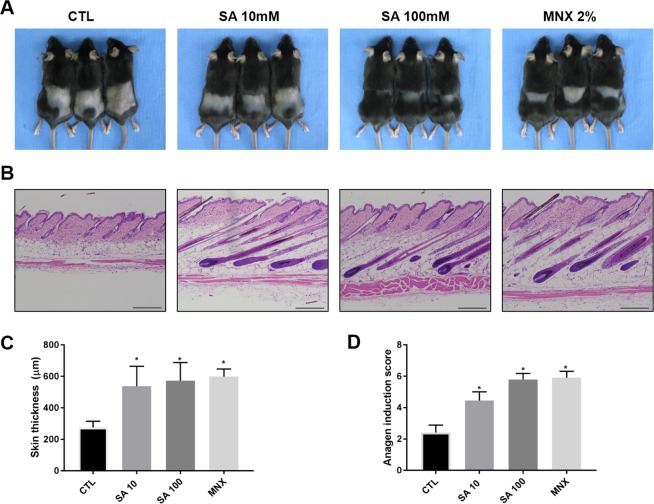
Effect of SA on anagen induction in 7-week-old female C57BL/6 mice. (**A**) Each group (n = 6 in each group) was topically treated with vehicle, SA (10 and 100 mM), or 2% minoxidil for 3 weeks. (**B**) Skin samples were obtained for histological analysis of H&E stained paraffin sections. (**C**) Skin thickness was measured as the distance from top of the epidermis to bottom of subcutaneous fat. (**D**) Anagen induction scores were calculated and assigned score values (telogen = 1, anagen I-VI = 2–7). Results are expressed as the mean ± SE, *P < 0.05 *versus* the control group. Scale bar = 200 μm; Original magnification, 100x. CTL: control, SA: shikimic acid, MNX: minoxidil.

To determine whether SA can enhance hair growth *in vivo*, we treated 7-week-old C57BL/6 female mice with topical SA. We topically applied vehicle, SA (10 and 100 mM), or 2% minoxidil to the shaved backs of mice (n = 6 in each study group) for 3 weeks. We found that the area of black skin was significantly larger in the SA-treated mice than in the control mice on day 21 (Fig. 1A). SA-treated mice did not show any evident adverse side effects. Histological studies showed that the thickness of interfollicular whole skin significantly increased in the SA- and MNX-treated mice (p < 0.05) (Figs. 1B,C). When anagen HFs were scored according to the anagen phase of each HFs, the score was significantly higher in the SA-treated group than in the control group (*P* < 0.05) (Fig. 1D).

### Mannose receptor (CD206) is expressed in human HFs

**Figure 2:**
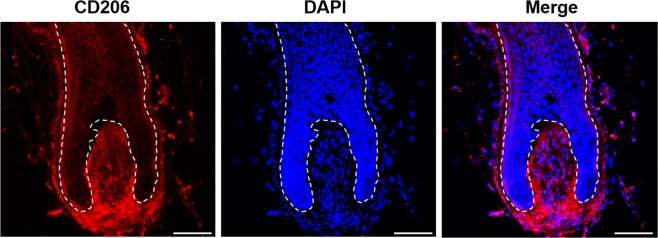
Expression of mannose receptor (MR, CD206) in human hair follicles. Immunofluorescent staining of MR was performed in hair follicles (red fluorescence for MR). Nuclei were stained using 4′,6-diamidino-2-phenylindole (DAPI, blue fluorescence). Scale bar = 100 μm.

We confirmed the expression of MR, which is a known receptor of SA, by performing immunofluorescence staining. In human HFs, MR was particularly highly expressed in the DP, as well as in the hair matrix keratinocytes (Fig. 2 and S1).

### SA enhances the viability of hDPCs and hORSCs

**Figure 3:**
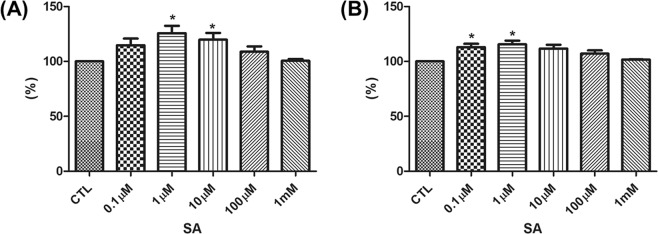
Effects of SA on the viability of cultured human dermal papilla cells (hDPCs) and outer root sheath cells (hORSCs). The proliferation of (**A**) hDPCs and (**B**) hORSCs was measured with an MTT assay after treatment of the cells with shikimic acid for 24 hours. Results are shown as mean ± SE (n = 5). *P < 0.05, *versus* the control group. CTL: control, SA: shikimic acid

To determine the effect of SA on HFs, we treated hDPCs and hORSCs for 24, 48 and 72 hours with various concentrations of SA and evaluated changes in cell proliferation by performing MTT assay. SA (1 and 10 µM) significantly enhanced the proliferation of hDPCs in comparison to that of vehicle-treated control at 24 h( *P* < 0.05) (Fig. 3A). Although hORSC proliferation was also enhanced by SA, the degree of increase was less than that of hDPCs (Fig. 3B). In addition, SA at dose 1, 10 and 100 µM significantly enhanced the proliferation of hDPCs compared to vehicle-treated control at 72 h (Fig. S2).

### SA enhances hair shaft elongation in *ex vivo* hair organ culture

**Figure 4:**
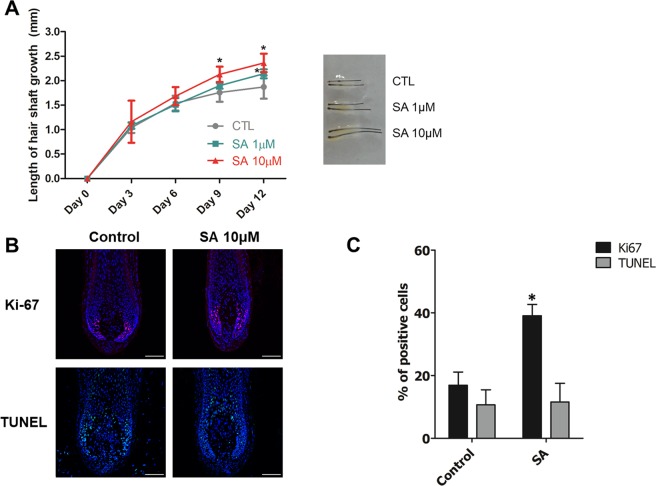
Effect of SA on hair shaft elongation in an *ex vivo* human hair follicle organ culture. (**A**) Human hair follicles (HFs) were treated with vehicle or SA (1 or 10 μM) for 12 days. SA enhanced hair shaft elongation compared the vehicle control. HF elongation was measured at 3, 6, 9, and 12 days. *Indicates the statistically significant difference (*P* < 0.05) between different groups using repeated-measured ANOVA analysis. (**B**) Human HFs were cultured *in vitro* with vehicle, SA 10 μM for 3 days and analyzed for proliferation (Ki67, red fluorescence) and apoptosis (TUNEL, green fluorescence) by using immunofluorescence staining. DAPI (blue fluorescence) was used to counterstain the nuclei. Scale bar = 100 μm. (**C**) For quantitative analysis, Ki67 + or TUNEL + cells were counted and normalized to DAPI-stained cells. Results are shown as mean ± SE (n = 5. *P < 0.05, *versus* the control group. CTL: control, SA: shikimic acid.

We performed *ex vivo* hair organ culture of human HFs to determine the effect of SA at the organ level. Human HFs were treated with 1 or 10 µM SA for 12 days, and elongation of the hair shaft was measured every third day. On day 9, HFs with 10 µM SA were found to be longer than HFs treated with vehicle, and on day 12, the HFs with 1 or 10 µM were significantly longer than those in the control group (*P* < 0.05, Fig. 4A and S3).

We also confirmed enhanced hair growth on the use of 10 µM SA by performing immunofluorescence staining. HFs cultured with 10 µM SA for 3 days were used to analyze proliferation (Ki-67, red fluorescence) and apoptosis (TUNEL, green fluorescence) in the hair matrix area; the effect was quantified by counting the Ki-67 + and TUNEL + cells and normalizing to DAPI-stained cells. SA treatment significantly increased the number of Ki-67 + cells, which accounted for 16.9% of the vehicle treated hair matrix and 39.1% of the 10 µM SA treated hair matrix (*P* < 0.05, Fig. 4B,C). Compared with the vehicle-treated group, the SA-treated group did not show a statistically significant change in the number of TUNEL + apoptotic cells (Fig. 4B,C).

### SA increases the expression of cytokines related to hair growth

**Figure 5:**
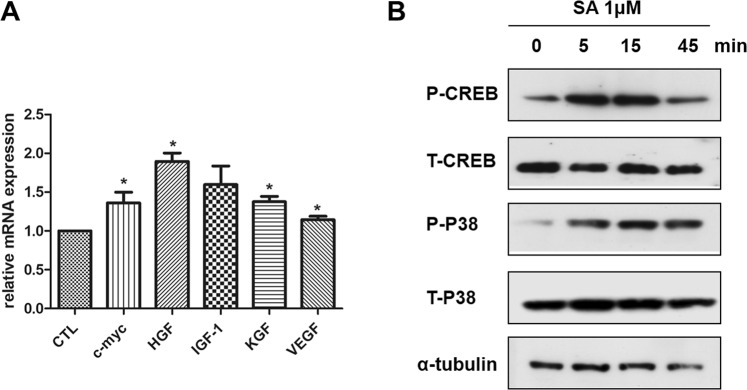
Effect of SA on the expression of cytokines and signaling molecules. (**A**) Human DPCs were treated with SA 1 μM for 24 hours, total RNA was isolated, and mRNA expression was analyzed using quantitative real time-PCR. The results are shown as means ± SE (n ≥ 3). *P < 0.05 versus the control. (**B**) Human DPCs treated with SA 1 μM were lysed and analyzed with Western blotting using primary antibodies against total-P38, phospho-P38, total-CREB, phospho-CREB, and α-tubulin. A representative data from three independent experiments are shown. CTL: control, DPCs: dermal papilla cells, SA: shikimic acid HGF: hepatocyte growth factor, IGF-1: insulin-like growth factor -1, KGF: keratinocyte growth factor, VEGF: vascular endothelial growth factor.

To evaluate the changes in the expression of growth factors, real-time PCR was performed using hDPCs treated with 1 μM SA for 24 hours. The expression levels of c-myc, HGF, KGF and VEGF in hDPCs increased significantly after SA treatment ( *P* < 0.05, Fig. 5A).

### SA activates the mitogen-activated protein kinase (MAPK) signaling pathway

Next, we performed western blotting to investigate the mechanism by which SA stimulates hair growth. We analyzed the expression of the various signaling molecules involved in cell proliferation, such as p38 and CREB in 1 μM SA-treated hDPCs. We found that p38 and CREB were phosphorylated 15 minutes after SA treatment in hDPCs (Fig. 5B).

## Discussion

To the best of our knowledge, this is the first work to show that SA promotes hair growth in both an *in vivo* mouse model and *in vitro* human HFs. Hair is an important element in determining the first impression of a person, and hair loss is an important issue from the viewpoint of quality of life. To date, proven treatments for hair loss have been limited to topical minoxidil and oral finasteride^24^. Recently, stem cells have been known to secrete various growth factors that affect hair growth, due to which they have been spotlighted as a novel therapeutic method for hair loss^3,4,25^. Since hair loss requires long-term treatment, the disadvantage is that conventional stem cell treatment of animal origin cannot be used easily. Therefore, it is necessary to develop a new treatment method that has fewer side effects and can be easily applied. From this viewpoint, hair loss treatment using SA, a plant stem cell extract, has advantages in terms of accessibility, safety, and efficacy.

SA is a key hydroaromatic intermediate in the essential aromatic amino acid biosynthesis and is known to be extracted from various plants^7,8^. There have been many studies on the various pharmacological effects of SA; however, there was no study on the effect of topical application of SA on human HFs. SA is expected to be easily absorbed through the skin owing to its low molecular weight (174.15 g/mol). Moreover, it is considered that topical SA does not have any significant side effects. As SA has been found to be a major component of plant stem cells, it can be used in a wide range of fields based on various effects such as secretion of growth factors and regeneration ability of stem cells^9^.

The hair growth-promoting effect of SA has been previously reported in a mouse organ culture model^12^. In this study, we confirmed the hair growth promoting effect of SA by performing an anagen induction assay in a C57BL/6 mice *in vivo* model. Hair growth was more prominently observed in the SA-treated group than in the vehicle-treated group, without side effects. A higher number of HFs and thicker skin were seen in SA-and MNX treated mice than in vehicle-treated mice. These findings suggest that topical SA effectively enhances hair growth.

To determine whether the same effect is observed in human HFs, we first needed to determine whether MR is expressed in human HFs. MR is a carbohydrate-binding receptor; in the skin, MR is expressed in keratinocytes and fibroblast. When MR is activated, it can trigger a protective pathway against autoimmune diseases^26^. MR selectively recognizes mannose, N-acetyl glucosamine, and fucose residue^13,14^. SA acts as a mannose bioisostere and interacts with the cell surface MR^5,20^. We observed MR expression in human HFs, especially hDP, by performing immunofluorescence staining. Furthermore, we demonstrated that SA significantly increased the proliferation of hDPCs and hORSCs, via an MTT assay. HF structure consists of DP, a mesenchymal cell cluster, and the surrounding matrix keratinocyte^27^. The interaction between DP and the surrounding cells plays an important role in hair growth and regeneration^27^. It is well known that DP plays a crucial role in HF maintenance and hair growth^22,27^. In this study, MR, a known SA receptor, was expressed more in DP than in the hair matrix keratinocyte, and the effect of SA on cell proliferation was more prominent in hDPCs than in hORSCs. Taken together, it is thought that SA particularly affects the DP and thus has a hair growth promoting effect. In this study, we first confirmed that MR is present in the HFs. Further research is needed on the mechanism of hair growth through MR, but this study is meaningful as it confirms that MR can be a new target of hair growth promoting therapy.

Next, we studied whether SA enhances hair shaft elongation in *ex vivo* human hair organ culture. It was found that SA significantly increased Ki-67+ cells in the hair matrix area, thus verifying the increased proliferation by SA. Our study also demonstrated that SA treatment for hDPCs significantly increased mRNA expression of c-myc, HGF, KGF and VEGF, which are known to stimulate hair growth^28–30^. Previous studies reported that c-myc activates stem cells in the HF and affects hair cycling by promoting the switch from telogen to anagen^29^. HGF, KGF and VEGF are also well known as a hair growth promoting factor, and we confirmed increased expression of these growth factors in hDPCs treated with SA^28,30^. Previously, adipose tissue-derived stem cells were seen to have various cytokine-secreting properties such as KGF, HGF, and VEGF, which were effective in hair growth^3,25^. In this study, SA, a plant stem cell extract, was also proved to have similar effects.

We investigated the molecular mechanism through which SA promotes hair growth. SA is known to interact with MR on the cell surface, which could activate myeloid differentiation primary response 88 (MyD88)^9,13,14^. The downstream pathway of MyD88 is known as p38 MAPK and CREB, which are known to promote cell survival and prevent apoptosis^31,32^. p38 MAPK phosphorylation can regulate the Wnt/β-catenin signaling pathway, which plays a pivotal role in the hair cycle^31^. The Wnt protein also induces phosphorylation of CREB and stimulates CREB-mediated transcription^31,32^. Since we demonstrated that SA increased the phosphorylation of p38 and CREB, we concluded that SA could enhance hair growth via MAPK-related signals, especially p38 and CREB.

## Conclusion

In summary, we found that SA promotes hair growth *in viv*o in a mouse model and *in vitro* in human HFs. These effects might be mediated through increase of growth factor levels and activation of the p38 MAPK-CREB pathway. Taken together, our results suggest that SA could be an important potential therapeutic agent for hair loss.

## Supplementary information

supplementary material

Western film original file

## Data Availability

All the datasets from the present study may be obtained from the corresponding author upon request.
