# Supplementary material for: Shikimic acid, a mannose bioisostere, promotes hair growth with the induction of anagen hair cycle
Source: Sci Rep. 2019 Nov 18;9:17008. doi: 10.1038/s41598-019-53612-5 (PMC6861222; doi:10.1038/s41598-019-53612-5)
Supplement: Supplementary file 1 — supplementary material [file 41598_2019_53612_MOESM1_ESM.docx]

**Supplementary Material**

**Shikimic acid, a mannose bioisostere, promotes hair growth with the induction of anagen hair cycle**

Mira Choi^a,1^, Soon-Jin Choi^b,c,d,1^, Sunhyae Jang^b,c,d^, Hye-In Choi^b,c,d^, Bo-Mi Kang^b,c,d^,

Sungjoo Tommy Hwang^e^, Ohsang Kwon^b,c,d,*^

^a^Department of Dermatology, College of Medicine, Ilsan Paik Hospital, Inje University, Gyeong-gi, Republic of Korea

^b^Department of Dermatology, College of Medicine, Seoul National University, Seoul, Republic of Korea, Republic of Korea

^c^Institute of Human-Environment Interface Biology, Medical Research Center, Seoul National University, Seoul, Republic of Korea

^d^Laboratory of Cutaneous Aging and Hair Research, Biomedical Research Institute, Seoul National University Hospital, Seoul, Republic of Korea

^e^Dr. Hwang's Hair-Hair Clinic, Seoul, Korea

**Legends for Supplementary Tables and Figures**

**Table S1. Primer sequences for qRT-PCR**

| **Gene** | **Forward Primer** | **Reverse Primer** |
| --- | --- | --- |
| c-myc | CCGCTTCTCTGAAAGGCTCT | AAGCTAACGTTGAGGGGCAT |
| HGF | AGCAGTCCTCGTAAGGCA | AATCTGCCTGGAAACACC |
| IGF-1 | ACGGCTGGACCGGAGACGGTC | CTACTTGCGTTCTTCAAATGT |
| KGF | TTGTGGCAATCAAAGGGGTG | CCTCCGTTGTGTGTCCATTTAGC |
| VEGF | CCATGAACTTTCTGCTGTCTT | TCGATCGTTCTGTATCAGTCT |
| 36B4 | TGGGCTCCAAGCAGATGC | GGCTTCGCTGGCTCCCAC |


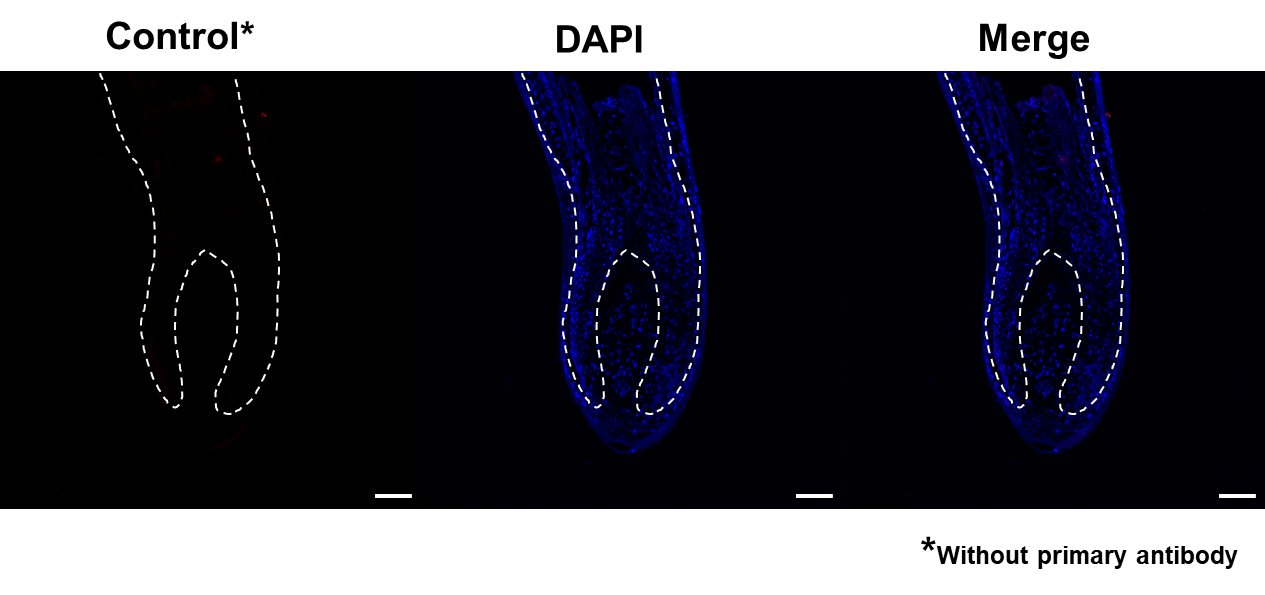
 **Fig. S1. Negative control staining for mannose receptor (MR, CD206) in human hair follicles.**

Immunofluorescent staining of negative control for MR was performed in hair follicles (red fluorescence without primary antibody (MR, CD 206)). Nuclei were stained using 4’,6-diamidino-2-phenylindole (DAPI, blue fluorescence). Scale bar =100 μm.

**
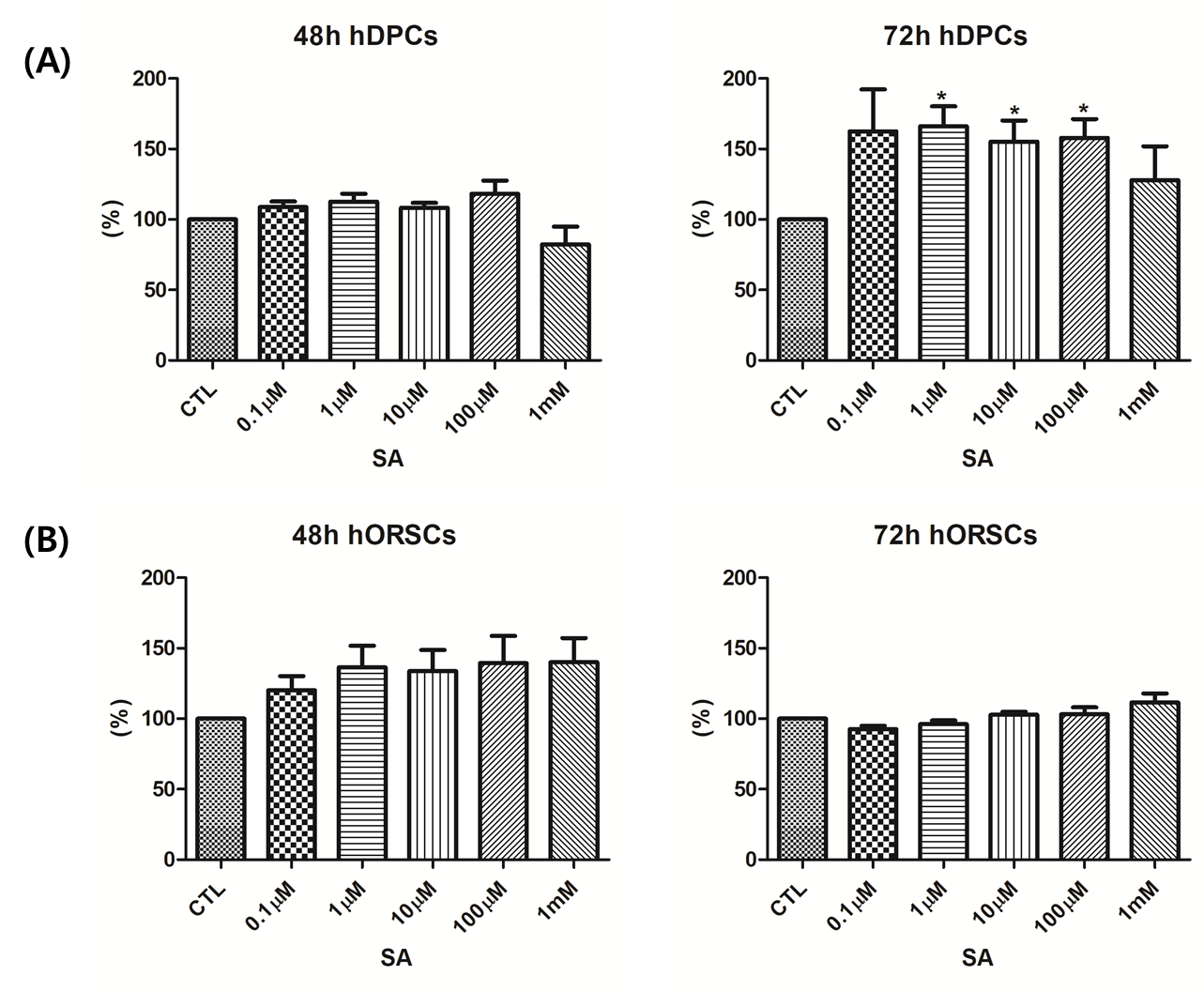
**

**Fig. S2. Effects of SA on the viability of cultured human dermal papilla cells (hDPCs) and outer root sheath cells (hORSCs).**

The proliferation of (A) hDPCs and (B) hORSCs was measured with an MTT assay after treatment of the cells with shikimic acid for 48 and 72 hours. Results are shown as mean± SE (n=5). **P*<0.05, *versus* the control group. CTL: control, SA: shikimic acid

**
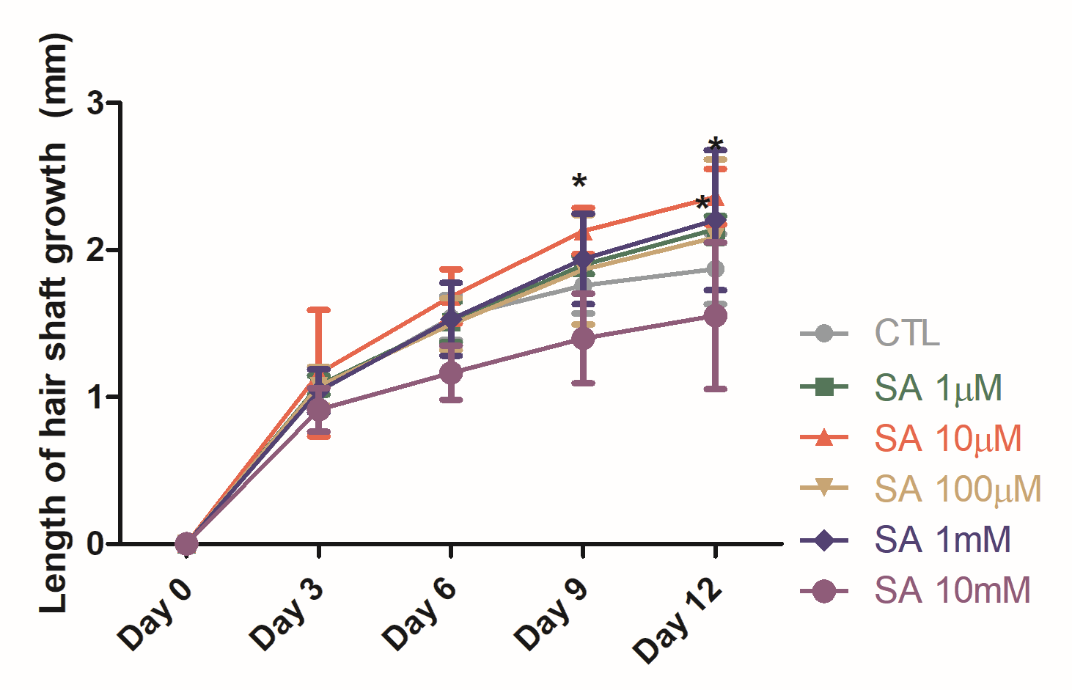
**

**Fig. S3. Effect of SA on hair shaft elongation in an *ex vivo* human hair follicle organ culture**

Human hair follicles (HFs) were treated with vehicle or SA (1, 10, 100 μM, 1 and 10mM) for 12 days. SA enhanced hair shaft elongation compared the vehicle control. HF elongation was measured at 3, 6, 9, and 12 days. * indicates the statistically significant difference (*P* < 0.05) between different groups using repeated-measured ANOVA analysis.

CTL: control, SA: shikimic acid
