# Supplementary material for: Shikimic acid, a mannose bioisostere, promotes hair growth with the induction of anagen hair cycle
Source: Sci Rep. 2019 Nov 18;9:17008. doi: 10.1038/s41598-019-53612-5 (PMC6861222; doi:10.1038/s41598-019-53612-5)
Supplement: Supplementary file 2 — Western film original file [file 41598_2019_53612_MOESM2_ESM.docx]

**Supplementary Material**

**Shikimic acid, a mannose bioisostere, promotes hair growth with the induction of anagen hair cycle**

Mira Choi^a,1^, Soon-Jin Choi^b,c,d,1^, Sunhyae Jang^b,c,d^, Hye-In Choi^b,c,d^, Bo-Mi Kang^b,c,d^,

Sungjoo Tommy Hwang^e^, Ohsang Kwon^b,c,d,*^

^a^Department of Dermatology, College of Medicine, Ilsan Paik Hospital, Inje University, Gyeong-gi, Republic of Korea

^b^Department of Dermatology, College of Medicine, Seoul National University, Seoul, Republic of Korea, Republic of Korea

^c^Institute of Human-Environment Interface Biology, Medical Research Center, Seoul National University, Seoul, Republic of Korea

^d^Laboratory of Cutaneous Aging and Hair Research, Biomedical Research Institute, Seoul National University Hospital, Seoul, Republic of Korea

^e^Dr. Hwang's Hair-Hair Clinic, Seoul, Korea

**Supplementary Figure 1 for Figure 5B. Effect of SA on the expression of cytokines and signaling molecules (Western blot films)**
